# Hospital Utilization Among Rural Children Served by Pediatric Neurology Telemedicine Clinics

**DOI:** 10.1001/jamanetworkopen.2019.9364

**Published:** 2019-08-16

**Authors:** Parul Dayal, Celia H. Chang, William S. Benko, Brad H. Pollock, Stephanie S. Crossen, Jamie Kissee, Aaron M. Ulmer, Jeffrey S. Hoch, Leslie Warner, James P. Marcin

**Affiliations:** 1 Department of Pediatrics, University of California Davis Health, Sacramento; 2 Now with Genentech Inc, South San Francisco, California; 3 Department of Neurology, University of California Davis Health, Sacramento; 4 Department of Public Health Sciences, University of California Davis Health, Sacramento; 5 Department of Pediatrics, University of California Davis Health, Sacramento; 6 Center for Health and Technology, University of California Davis Health, Sacramento; 7 Center for Healthcare Policy and Research, University of California Davis Health, Sacramento; 8 Shasta Community Health Center, Redding, California

## Abstract

**Importance:**

Telemedicine is increasingly used to provide outpatient pediatric neurology consultations in underserved communities. Although telemedicine clinics have been shown to improve access, little is known about how they alter patients’ utilization of hospital services.

**Objective:**

To evaluate the association between access to telemedicine clinics and hospital utilization among underserved children with neurologic conditions.

**Design, Setting, and Participants:**

This retrospective cross-sectional study included 4169 patients who received outpatient care from pediatric neurologists affiliated with an academic children’s hospital in California between January 1, 2009, and July 31, 2017, either in person or using telemedicine.

**Exposures:**

Consultation modality (telemedicine or in person) in the outpatient neurology clinics.

**Main Outcomes and Measures:**

Demographic and clinical variables were abstracted from the hospital’s electronic medical records. The association between the modality of outpatient neurology care and patients’ utilization of the emergency department and hospitalizations was evaluated. Both all-cause and neurologic condition–related hospital utilization were analyzed using multivariable negative binomial regression in overall and matched samples.

**Results:**

The telemedicine cohort comprised 378 patients (211 [55.8%] male), and the in-person cohort comprised 3791 patients (2090 [55.1%] male). The mean (SD) age at the first encounter was 7.4 (5.4) years for the telemedicine cohort and 7.8 (5.1) years for the in-person cohort. The telemedicine cohort was more likely than the in-person cohort to have nonprivate insurance (public insurance, self-pay, or uninsured), lower education, and lower household income. The rates of all-cause and neurologic hospital encounters were lower among children who received pediatric neurology consultations over telemedicine compared with children who received care in the in-person clinics (5.7 [95% CI, 3.5-8.0] vs 20.1 [95% CI, 18.1-22.1] per 100 patient-years and 3.7 [95% CI, 2.0-5.3] vs 8.9 [95% CI, 7.8-10.0] per 100 patient-years, respectively; *P* < .001). Even after adjusting for demographic and clinical factors, the telemedicine cohort had a lower risk of hospital encounters (emergency department visits and admissions) with an adjusted incidence rate ratio of 0.57 (95% CI, 0.38-0.88) for all-cause encounters and an adjusted incidence rate ratio of 0.60 (95% CI, 0.36-0.99) for neurologic encounters. After matching on travel time to the neurology clinic, the adjusted incidence rate ratio was 0.19 (95% CI, 0.04-0.83) for all-cause admissions and 0.14 (95% CI, 0.02-0.82) for neurologic admissions.

**Conclusions and Relevance:**

Pediatric neurology care through real-time, audiovisual telemedicine consultations was associated with lower hospital utilization compared with in-person consultations, suggesting that high-cost hospital encounters can be prevented by improving subspecialty access.

## Key Points

**Question:**

What is the association between access to outpatient telemedicine consultations and hospital utilization among medically underserved children with neurologic conditions?

**Findings:**

This cross-sectional study of 4169 children found that the rate of hospital encounters among children who obtained pediatric neurology consultations using telemedicine was significantly lower than the rate among similar children who obtained in-person care.

**Meaning:**

By improving access to pediatric neurology consultations in underserved communities and enhancing care coordination, telemedicine may reduce the utilization of high-cost hospital services.

## Teaser

This cross-sectional study of pediatric neurology patients residing in rural areas compares rates of hospital encounters for patients who had outpatient telemedicine consultations vs those who visited neurology clinics in person.

## Introduction

Limited access to outpatient pediatric neurology care can lead to inconsistent management of patients’ medical conditions and may result in unplanned hospital encounters, including visits to the emergency department (ED) and hospital admissions.^1,2,3,4,5,6,7,8^ For children, appropriate access to outpatient care is hindered by the shortage of pediatric neurologists across the country.^9^ Confounding these shortages is the fact that pediatric subspecialists are concentrated at tertiary care centers located primarily in urban areas,^10,11,12^ forcing children with neurologic disorders living in rural communities to travel long distances to see the nearest pediatric neurologist.

Real-time telemedicine consultations may reduce the time and financial burden of subspecialty appointments for underserved patients.^1,13^ University of California Davis Children’s Hospital (UCDCH) has been providing outpatient pediatric neurology services through telemedicine to primary care clinics in underserved communities of California since 2009. In a recent study,^14^ we found that these pediatric neurology telemedicine appointments are more likely to be completed rather than canceled or missed (ie, no-show) compared with in-person appointments among a cohort of children with similar demographic characteristics and neurologic conditions. This and other similar studies^1,13,14^ suggest that outpatient telemedicine may improve access to subspecialty care for underserved populations. However, whether the increased access to care from telemedicine results in a reduction in hospital encounters is not well studied.^11,15,16,17,18^

To better understand how outpatient telemedicine models of care are associated with patients’ utilization of hospital services, we compared the rates of ED visits and hospital admissions at UCDCH between similar cohorts of patients who obtained outpatient pediatric neurology care either at the remote telemedicine clinics or at the on-site, in-person clinics.

## Methods

The institutional review board at UCDCH approved this study. Because the data were retrospectively collected and deidentified, a waiver from the requirement of informed consent was granted to this study. This study adhered to the Strengthening the Reporting of Observational Studies in Epidemiology (STROBE) reporting guideline.

### Telemedicine Model

Between January 1, 2009, and December 31, 2018, the Division of Pediatric Neurology at UCDCH completed more than 1000 telemedicine visits with patients in underserved and rural communities; this study analyzes visits that occurred between January 1, 2009, and July 31, 2017. Telemedicine consultations were offered for new and follow-up appointments at 13 remote sites in northern California. Remote clinic staff and primary care physicians collected each patient’s vital statistics and history, performed a detailed physical examination and recorded the findings, and discussed visit recommendations together with the patient and neurologist. Laboratory test results (eg, electroencephalography) and neurologic images (eg, computed tomography or magnetic resonance imaging) were faxed, mailed, or shared over picture archiving and communication systems to the pediatric neurologist either before or during the appointment. Live videoconferencing was conducted throughout the study using similar hardware, including turnkey telemedicine codecs with full UCDCH physician access to remote pan-tilt-zoom capabilities. The pediatric neurologist conducted the consultation in a telemedicine consultation room equipped with a desk, a computer with the electronic health record (EHR), dual computer monitors, and the wall-mounted monitor and videoconferencing unit. The pediatric neurologist then documented the consultation note within UCDCH’s EHR system, and this note was either electronically shared or faxed to the remote clinic site.

### Study Population and Outcome

The study population consisted of patients aged 18 years and younger whose registered home addresses were within UCDCH’s 33-county service area in northern California and who completed at least 1 clinic visit with a UCDCH pediatric neurologist between January 1, 2009, and July 31, 2017, either over telemedicine or in person. We did not include patients who were scheduled but never seen. For each included patient, time in the study or the observation period during which they were considered to be at risk for a hospital encounter (ED visit or hospital admission) was defined as the time between the patient’s first completed neurology appointment and the date when the patient turned 19 years old or July 31, 2017, whichever occurred first. The time at risk in the study for each patient excluded days during which the child was hospitalized. Hospital encounters that occurred within 24 hours of a previous discharge were not counted as an additional encounter, but were instead considered part of the prior hospital episode.

### Data Source and Variables

We abstracted demographic, clinical, and utilization data from the UCDCH EHR. It is important to note that during the years 2009 to 2017, no other hospitals in the communities included in our analyses had pediatric inpatient wards with the clinical capacity to care for children with neurologic conditions. In addition, we limited our analyses to patients in northern California, where there were no practicing pediatric neurologists using telemedicine outside of UCDCH. Therefore, it is a reasonable assumption that if pediatric patients cared for by UCDCH pediatric neurologists in the telemedicine or in-person clinics required hospital services, they would be transferred or admitted directly to UCDCH.

Sex, insurance status, and patient addresses were assumed to stay constant throughout the study period, and their values were designated as those recorded in the EHR at the time of data extraction. Insurance status was dichotomized into private (commercial employer-based) and nonprivate, which included public insurance (eg, Medicaid or managed Medicaid), self-pay, and no insurance. Addresses were geocoded and mapped to US Census tracts. Aggregate Census tract information was used to assign patients’ neighborhood median household income and education level (defined as the proportion of residents with a bachelor’s degree or higher) using the 2016 American Community Survey’s 5-year estimates.^19^ Geocoded addresses were used to estimate patients’ travel times to their outpatient neurology clinic (ie, time needed to travel from the patient’s home to the remote telemedicine clinic for the telemedicine cohort or to UCDCH for the in-person cohort), as well as patients’ travel time to UCDCH. Travel times were estimated using a proprietary geolocation application programming interface to compute travel distance and travel time between 2 points defined by their geographic coordinates, assuming motor vehicle speeds under standard traffic conditions.^20^

*International Classification of Diseases*,* Ninth Revision* and *International Classification of Diseases*,* Tenth Revision* codes for up to 5 encounter diagnoses were used to determine whether the hospital encounter was related to a neurologic condition using manual review of codes and applying previously published criteria.^21,22,23,24,25,26,27^ Neurologic conditions were grouped into clinically relevant categories for comparison between the cohorts. Because most patients included in the study did not have any hospital encounters, we also compared patients’ neurology clinic diagnoses between the cohorts. In addition, *International Classification of Diseases*,* Ninth Revision* and *International Classification of Diseases*,* Tenth Revision* diagnosis codes recorded during hospital and/or clinic encounters were used to determine whether the patient had a complex chronic condition using a previously validated algorithm.^28^

### Statistical Analysis

Simple descriptive statistics were used to characterize study variables. Univariable and bivariable comparisons were conducted using *t* tests, Pearson χ^2^ tests, and Wilcoxon rank sum tests, as appropriate. The primary outcome variable was hospital encounter rate, calculated as the total number of ED visits and hospital admissions per patient-year. Rates were calculated for hospital encounters related to any condition (all-cause) and those related to neurologic conditions. The primary independent variable was whether the patient received outpatient care in the telemedicine clinics or the in-person clinic. The telemedicine cohort included patients who scheduled 1 or more of their outpatient neurology appointments in a telemedicine clinic, and the in-person cohort included patients who scheduled all their appointments in the in-person clinics.

We compared the rate of all-cause and neurologic hospital encounters between the telemedicine and in-person cohorts by estimating the ratio of rates (incidence rate ratio [IRR]) using negative binomial regression. The negative binomial model allowed us to account for overdispersion in the total number of hospital encounters and was a better fit for our data than the Poisson model. The patient’s time in the study was used as an offset in the model. Models were adjusted for confounders, including insurance status, median household income, travel time to UCDCH, presence of a complex chronic condition, and outpatient neurology clinic diagnoses. The confounders were chosen for inclusion in the multivariable model on the basis of a priori assumptions, as well as the associations observed in the descriptive analysis. Outpatient neurology clinic diagnoses were collapsed into 3 broad categories according to clinical affinity for inclusion in the multivariable model. In an additional analysis, we included the patient’s outpatient neurology appointment completion rate in the adjusted multivariable model to determine how much of the differential risk of having a hospital encounter in the telemedicine cohort was associated with neurology clinic appointment adherence.

To check the robustness of our findings, we evaluated hospital encounter rates in matched subsets of the study population. First, we matched the telemedicine and in-person cohorts on travel time to UCDCH using a metric of 5 minutes in a 1:1 ratio (without replacement) to compare the rates among cohorts living in communities far from UCDCH. Second, we matched the cohorts on travel time to their neurology clinic (remote telemedicine clinics for the telemedicine cohort and in-person clinics at UCDCH for the in-person cohort) using the same method already described to compare hospital encounter rates among cohorts with similar access to outpatient neurology care, with respect to travel time. For this analysis, we limited the outcome to inpatient admissions because ED visits without hospitalization would be more likely to occur at UCDCH among the in-person cohort at baseline, independent of the exposure.

All analyses were conducted using Stata/SE statistical software version 15.1 (StataCorp). Two-sided *P* < .05 was considered statistically significant.

## Results

A total of 4169 patients with at least 1 completed appointment with a UCDCH pediatric neurologist between January 1, 2009, and July 31, 2017, were included in the study. Of these, 378 patients (9.1%) were included in the telemedicine cohort (211 [55.8%] male), and 3791 patients (90.9%) were included in the in-person cohort (2090 [55.1%] male) (Table 1). The mean (SD) age at the first encounter was 7.4 (5.4) years for the telemedicine group and 7.8 (5.1) years for the in-person group. Thirty-nine patients had appointments in both telemedicine and in-person clinics and were included in the telemedicine cohort.

**Table 1.** Distribution of Baseline Characteristics Among Telemedicine and In-Person Cohorts

Characteristic	Telemedicine Cohort	In-Person Cohort	*P* Value
Total, No. (%)	378 (9.1)	3791 (90.9)	
Age at first encounter, mean (SD), y	7.4 (5.4)	7.8 (5.1)	.16
Sex, No. (%)			
Female	167 (44.2)	1701 (44.9)	.80
Male	211 (55.8)	2090 (55.1)	
Insurance, No. (%)			
Private	8 (2.1)	1387 (36.6)	<.001
Nonprivate (public, self-pay, or other)	370 (97.9)	2404 (63.4)	
Median household income, mean (SD), US $	42 600 (12 600)	69 300 (29 600)	<.001
Parents with bachelor’s degree or higher, mean (SD), %^a^	17.5 (7.4)	31.3 (17.9)	<.001
Travel time to neurology clinic, mean (SD), min	20.6 (24.4)	48.0 (52.4)	<.001
Travel time to University of California Davis Children’s Hospital, mean (SD), min	156.0 (33.9)	48.0 (52.4)	<.001
Clinic appointments completed, mean (SD), %	81.7 (23.5)	75.7 (25.4)	<.001
Time in study per patient, y			
Mean (SD)	3.6 (2.2)	4.5 (2.7)	<.001
Median (IQR)	3.1 (1.8, 5.3)	4.6 (2.2, 7.0)	
Patients with a complex chronic condition, No. (%)			
No	317 (83.9)	3290 (88.8)	.18
Yes	52 (13.8)	446 (11.8)	
Missing data	9 (2.4)	55 (1.5)	
Neurology clinic diagnosis, No. (%)			
Seizures and suspected seizures	137 (36.2)	1120 (29.5)	<.001
Developmental disorders	47 (12.4)	594 (15.7)	
Headaches and migraine	25 (6.6)	615 (16.2)	
Disorders of muscle and nerve^b^	48 (12.7)	453 (12.0)	
Genetic and congenital disorders	29 (7.7)	192 (5.1)	
Cerebral degeneration, damage, or injury	13 (3.4)	160 (4.2)	
Other low-severity disorders^c^	33 (8.7)	419 (11.1)	
General or nonspecific	37 (9.8)	173 (4.5)	
Missing	9 (2.4)	65 (1.7)	
Patients with ≥1 all-cause hospital encounter, No. (%)	40 (10.6)	1024 (27.0)	<.001
Patients with ≥1 neurologic hospital encounter, No. (%)	28 (7.4)	473 (12.5)	.004

Abbreviation: IQR, interquartile range.

^a^ In patient’s US Census tract region.

^b^ Including movement disorders.

^c^ Including fatigue, sleep, vision, infection, neoplasm, behavioral, mental, social, skin, ear, or hearing disorders.

As shown in Table 1, the cohorts had comparable distributions of age and sex; however, patients in the telemedicine cohort were less likely to have private insurance compared with patients in the in-person cohort (2.1% vs 36.6%). Patients in the telemedicine cohort were also more likely to live in regions with a lower median household income (mean [SD], $42 600 [$12 600] vs $69 300 [$29 600]) and lower education level (mean [SD], 17.5% [7.4%] vs 31.3% [17.9%] college graduates). The mean (SD) travel time for outpatient neurology care was 20.6 (24.4) minutes for the telemedicine cohort, and 48.0 (52.4) minutes for the in-person cohort. In contrast, the mean (SD) travel time to the UCDCH in-person clinic was 156.0 (33.9) minutes for the telemedicine cohort. The telemedicine cohort had a higher neurology clinic appointment completion rate than the in-person cohort (mean [SD], 81.7% [23.5%] vs 75.7% [25.4%]). There were differences in the distribution of neurology clinic diagnoses between the cohorts (Table 1); however, the distributions of patients with complex chronic conditions (Table 1) and neurologic hospital diagnoses (eTable 1 in the Supplement) were comparable between the 2 cohorts.

In terms of hospital encounters, 40 telemedicine patients (10.6%) (Table 1) had 77 all-cause hospital encounters (Table 2), and 28 telemedicine patients (7.4%) (Table 1) had 49 neurologic hospital encounters (Table 2). In comparison, 1024 in-person patients (27.0%) (Table 1) had 3455 all-cause hospital encounters (Table 2), and 473 in-person patients (12.5%) (Table 1) had 1531 neurologic hospital encounters (Table 2). Frequencies and rates of hospital encounters for each cohort and encounter type are shown in Table 2. The rate of all-cause hospital encounters was lower among children who received pediatric neurology consultations over telemedicine compared with children who received care by traveling to the clinic (5.7 [95% CI, 3.5-8.0] vs 20.1 [95% CI, 18.1-22.1] per 100 patient-years, respectively; *P* < .001). The rate of hospital encounters for neurologic-related reasons was also lower among the telemedicine cohort compared with the in-person cohort (3.7 [95% CI, 2.0-5.3] vs 8.9 [95% CI, 7.8-10.0] per 100 patient-years, respectively; *P* < .001) (Table 2).

**Table 2.** Hospital Encounter Frequencies and Rates by Cohort

Hospital Encounter Type	Telemedicine Cohort		In-Person Cohort	
	Patients, No.	No. of Encounters/100 Patient-Years (95% CI)^a^	Patients, No.	No. of Encounters/100 Patient-Years (95% CI)^b^
All-cause encounters	77	5.7 (3.5-8.0)	3455	20.1 (18.1-22.1)
Emergency department visits	9	0.7 (0.0-1.4)	1966	11.4 (10.3-12.6)
Hospital admissions	68	5.1 (2.9-7.2)	1489	8.7 (7.5-9.8)
Neurologic encounters	49	3.7 (2.0-5.3)	1531	8.9 (7.8-10.0)

^a^ Total 1341.8 patient-years in the cohort.

^b^ Total 17 205.6 patient-years in the cohort.

As shown in Table 3, the bivariable all-cause and neurologic hospital encounter rates were lower for patients in the telemedicine cohort compared with patients in the in-person cohort (for all-cause encounters, IRR, 0.25; 95% CI, 0.18-0.36; for neurologic encounters, IRR, 0.35; 95% CI, 0.23-0.54). Hospital encounter rates were inversely associated with the patient’s travel time to the neurology clinic (telemedicine or in person), median household income, education level, and completion rate of neurology clinic appointments. Rates of hospital encounters were higher for patients who had nonprivate insurance than for patients who had private insurance (Table 3).

**Table 3.** Bivariable and Unadjusted Association of Hospital Encounter Rate With Patient Factors

Patient Factors	Incidence Rate Ratio (95% CI)^a^	
	All-Cause Encounters	Neurologic Encounters
Cohort		
In-person	1 [Reference]	1 [Reference]
Telemedicine	0.25 (0.18-0.36)	0.35 (0.23-0.54)
Age at first encounter, y	1.00 (0.98-1.01)	1.01 (0.99-1.03)
Sex, No. (%)		
Female	1 [Reference]	1 [Reference]
Male	1.14 (0.96-1.35)	1.02 (0.83-1.26)
Insurance, No. (%)		
Private	1 [Reference]	1 [Reference]
Nonprivate (public, self-pay, or other)	1.36 (1.14-1.63)	1.51 (1.20-1.90)
Travel time to neurology clinic, h	0.65 (0.58-0.72)	0.75 (0.64-0.85)
Travel time to University of California Davis Children’s Hospital, h	0.59 (0.54-0.65)	0.68 (0.60-0.76)
Median household income (per $10 000)^b^	0.90 (0.88-0.93)	0.88 (0.85-0.92)
Bachelor’s degree or higher (per 10% college graduates)^b^	0.85 (0.81-0.89)	0.80 (0.75-0.84)
Neurology clinic appointments completed, per 10%	0.94 (0.90-0.97)	0.90 (0.86-0.94)
Presence of a complex chronic condition		
No	1 [Reference]	1 [Reference]
Yes	1.16 (0.90-1.51)	1.61 (1.19-2.20)
Neurology clinic diagnosis category		
Seizures, developmental disorders and cerebral degeneration, damage, or injury	1 [Reference]	1 [Reference]
Disorders of the muscle and nerve and genetic and congenital disorders	0.86 (0.68-1.08)	0.72 (0.54-0.95)
Headaches and other low-severity disorders^c^	0.53 (0.43-0.64)	0.35 (0.27-0.44)

^a^ Incidence rate ratio from negative binomial regression with patient’s time in the study (years) as an offset.

^b^ In patient’s US Census tract region.

^c^ Including migraine, fatigue, sleep, vision, infection, neoplasm, behavioral, mental, social, skin, ear, and hearing disorders and general symptoms.

In the adjusted analysis (Table 4), rates of hospital encounters were lower for the telemedicine cohort compared with the in-person cohort (for all-cause encounters, adjusted IRR [aIRR], 0.57; 95% CI, 0.38-0.88; for neurologic encounters, aIRR, 0.60; 95% CI, 0.36-0.99). Hospital encounter rates were higher for patients with nonprivate insurance and were inversely associated with travel time to UCDCH. Patients who sought neurology clinic appointments for headaches and other disorders were less likely to have hospital encounters than those who sought appointments for seizure disorders, developmental disorders, and cerebral impairment (Table 4).

**Table 4.** Multivariable Model Showing the Association of Hospital Encounter Rate With Patient Factors

Variable	Adjusted Incidence Rate Ratio (95% CI)^a^	
	All-Cause Encounters	Neurologic Encounters
Cohort		
In-person	1 [Reference]	1 [Reference]
Telemedicine	0.57 (0.38-0.88)	0.60 (0.36-0.99)
Insurance status		
Private	1 [Reference]	1 [Reference]
Nonprivate (public, self-pay, or other)	1.08 (0.89-1.31)	1.16 (0.91-1.46)
Median household income (per $10 000)^b^	0.89 (0.87-0.92)	0.88 (0.84-0.91)
Travel time to University of California Davis Children’s Hospital, h	0.58 (0.52-0.66)	0.66 (0.57-0.76)
Pediatric complex chronic condition		
No	1 [Reference]	1 [Reference]
Yes	1.14 (0.88-1.48)	1.49 (1.10-2.01)
Neurology clinic diagnosis category		
Seizures, developmental disorders, and cerebral degeneration, damage, or injury	1 [Reference]	1 [Reference]
Disorders of the muscle and nerve and genetic and congenital disorders	0.93 (0.74-1.16)	0.75 (0.57-0.99)
Headaches and other low-severity disorders^c^	0.55 (0.45-0.67)	0.37 (0.29-0.48)

^a^ Adjusted incidence rate ratio from negative binomial regression with patient’s time in the study (years) as an offset.

^b^ In patient’s US Census tract region.

^c^ Including migraine, fatigue, sleep, vision, infection, neoplasm, behavioral, mental, social, skin, ear, and hearing disorders and general symptoms.

The rate of all-cause hospital encounters in the multivariable model, also adjusted for the percentage of neurology clinic appointments completed by the patient, was lower for the telemedicine cohort compared with the in-person cohort (aIRR, 0.59; 95% CI, 0.38-0.90). In addition, completion of outpatient appointments was inversely proportional to the hospital encounter rate (6% [95% CI, 3%-9%] lower all-cause hospital encounter rate and 9% [95% CI, 5%-13%] lower neurologic encounter rate for a 10% increase in the outpatient appointments completion rate). Excluding the 39 patients who had both telemedicine and in-person appointments and retaining the telemedicine-only and in-person-only cohorts in the analysis did not change our finding that the hospital encounter rates were lower in the telemedicine cohort (eTable 2 in the Supplement).

As shown in eTable 3 in the Supplement, the sample matched on travel time to UCDCH comprised 187 patients in each cohort. Bivariable and adjusted rates of hospital encounters were comparable between the cohorts. The sample matched on travel time to a neurology clinic comprised 378 patients in each cohort. The adjusted rates of all-cause and neurologic hospital admissions were lower among the telemedicine cohort than the in-person cohort (for all-cause admissions, aIRR, 0.19; 95% CI, 0.04-0.83; for neurologic admissions, aIRR, 0.14; 95% CI, 0.02-0.82).

## Discussion

In this retrospective cross-sectional study, we found that the rate of all-cause hospital encounters (ie, ED visits or hospital admissions) was approximately 4 times lower among children who received pediatric neurology consultations over telemedicine in their local communities compared with children who received care by traveling to the academic, urban, in-person pediatric neurology clinic (5.7 [95% CI, 3.5-8.0] vs 20.1 [95% CI, 18.1-22.1] per 100 patient-years; *P* < .001). We also found that the rate of hospital encounters for neurologic-related reasons was almost twice as low among the telemedicine cohort compared with the in-person cohort (3.7 [95% CI, 2.0-5.3] vs 8.9 [95% CI, 7.8-10.0] per 100 patient-years; *P* < .001). Our finding of lower hospital utilization among the telemedicine cohort remained significant and consistent even after adjusting for insurance status, median household income, travel time to UCDCH, neurology clinic diagnoses, and the presence of a complex chronic condition.

Our findings are consistent with previous studies that have found that improving access to outpatient care may prevent avoidable utilization of hospital services. For example, pediatric primary care telemedicine at schools and childcare centers improved access to care and resulted in a reduction in ED utilization compared with usual care.^16,29^ Within neurology specifically, poor access to outpatient care attributable to longer-than-average wait times was associated with a 7-fold higher likelihood of an ED visit, and reducing wait times by setting up urgent care clinics was associated with a reduction in seizure-related ED visits among children.^6,7^ In our study, the lower rate of hospital encounters in the telemedicine cohort compared with the in-person cohort is in agreement with these findings.

The lower rate of hospital utilization in the telemedicine cohort could be attributed to the higher completion rate of neurology appointments, resulting in better management of patients’ medical conditions, which may reduce the number of hospitalizations. However, we found a significant but small independent association between appointment completion and the hospital encounter rate in the adjusted analysis. This finding suggests that other factors also explain the lower hospital utilization among the telemedicine cohort. Such factors may include improved care coordination between the child’s primary care physician and neurologist in the telemedicine clinics, which facilitates the exchange of important health information between the physicians and parents and broadens the primary care physician’s knowledge about management of the patient’s neurologic condition. The need for care coordination tends to be greater for children with chronic conditions such as epilepsy and seizure disorders, which often confer developmental and mental health comorbidities and functional limitations,^15,30^ making the treatment of such children more appropriate for team-based care. Moving the system of care closer to a patient’s medical home may increase the quality of the care process, resulting in better outcomes.^31,32,33,34^

Lower hospital utilization among the telemedicine cohort could also be explained by the higher mean travel time to UCDCH for patients who use telemedicine compared with patients who normally travel to UCDCH for in-person outpatient care. Although our multivariable analysis adjusted for travel time to UCDCH found significantly lower rates of hospital utilization in the telemedicine cohort, we did not find significantly lower rates of hospital utilization in the telemedicine cohort among the patients matched on travel time to UCDCH. This nonsignificant finding, however, may be associated with a small sample size lacking sufficient power to detect a statistically significant difference.

### Limitations

This retrospective analysis has several limitations. First, there are inherent differences between the cohorts because patients were not randomized to telemedicine or in-person clinics. However, we attempted to address this limitation by using a multivariable model to adjust for potential confounders. Second, because we did not have access to patients’ medical records from other community hospitals in UCDCH’s service area, some patients might have had encounters at other hospitals that were not captured in our data. Thus, hospital rates for patients residing in distant communities could be underestimated in our study. We attempted to address this limitation by matching the cohorts on travel time to UCDCH (comparable risk of UCDCH hospital utilization) and restricting our comparison to inpatient admissions among patients matched on travel time to neurology clinics (comparable access to outpatient neurology care and comparable risk of admissions at UCDCH). However, whether the risk of a hospital encounters is comparable in the matched analyses would need to be supported with additional data in future studies. Third, patients were able to obtain telemedicine or in-person consultations at neurology clinics throughout the observation period for hospital encounters; thus, the outcome did not always temporally follow the exposure. However, in this analysis, our main exposure was the overall model of outpatient care and not the consultation modality for each individual outpatient appointment. Fourth, we were not able to determine whether admissions were planned or unplanned; hence, some of the hospital encounters captured may represent planned admissions (eg, for electroencephalogram monitoring or medication titration) as a direct result of subspecialty outpatient management. Fifth, our findings may not extend to patients who were referred but not scheduled or patients who were scheduled but never saw a pediatric neurologist, because these patients were excluded from our analysis.

## Conclusions

We found lower rates of hospital encounters among children who received neurology care in their own communities using telemedicine compared with children who received neurology care in the in-person clinics, even in multivariable analysis and certain matched analyses. Our findings suggest that by improving subspecialty access in underserved communities and enhancing care coordination among physicians, telemedicine may reduce the utilization of high-cost hospital care for children with neurologic conditions.

## Notes

Supplement. **eTable 1.** Comparison of Diagnosis Categories Among Neurological Hospital Encounters **eTable 2.** Sensitivity Analysis Excluding 39 Patients Who Had Both Telemedicine and In-Person Neurology Appointments **eTable 3.** Comparison of Hospital Encounter Frequencies and Rates in Matched Telemedicine and In-Person Patient Cohorts
