# Supplementary material for: Hospital Utilization Among Rural Children Served by Pediatric Neurology Telemedicine Clinics
Source: JAMA Netw Open. 2019 Aug 16;2(8):e199364. doi: 10.1001/jamanetworkopen.2019.9364 (PMC6704740; doi:10.1001/jamanetworkopen.2019.9364)
Supplement: Supplement. — eTable 1. Comparison of Diagnosis Categories Among Neurological Hospital Encounters eTable 2. Sensitivity Analysis Excluding 39 Patients Who Had Both Telemedicine and In-Person Neurology Appointments eTable 3. Comparison of Hospital Encounter Frequencies and Rates in Matched Telemedicine and In-Person Patient Cohorts [file jamanetwopen-2-e199364-s001.pdf]

Supplementary Online Content

Dayal P, Chang CH, Benko WS, et al. Hospital utilization among rural children served by pediatric neurology telemedicine clinics. *JAMA Netw Open*. 2019;2(8):e199364. doi:10.1001/jamanetworkopen.2019.9364

eTable 1. Comparison of Diagnosis Categories Among Neurological Hospital Encounters
eTable 2. Sensitivity Analysis Excluding 39 Patients Who Had Both Telemedicine and In-Person Neurology Appointments
eTable 3. Comparison of Hospital Encounter Frequencies and Rates in Matched Telemedicine and In-Person Patient Cohorts

This supplementary material has been provided by the authors to give readers additional information about their work.

© 2019 Dayal P et al. *JAMA Network Open.*

eTable 1. Comparison of Diagnosis Categories Among Neurological Hospital Encounters[a]

Neurological Diagnosis Category	Total, N (%)	Telemedicine cohort, N (%)	In-person cohort, N (%)
Seizures and suspected seizures	768 (48.6)	29 (59.2)	739 (48.3)
Developmental delays and behavioral/mental/social disorders	190 (12.0)	7 (14.3)	183 (12.0)
Other low-severity[b]	178 (11.3)	3 (6.1)	175 (11.4)
Disorders affecting the muscle and nerves, including movement disorders	150 (9.5)	5 (10.2)	145 (9.5)
Cerebral degeneration and inflammation, spinal cord inflammation & other brain disorders	121 (7.7)	2 (4.1)	119 (7.8)
Headaches and migraines	99 (6.3)	2 (4.1)	97 (6.3)
Intracranial/cerebral/spinal cord injury or damage	74 (4.7)	1 (2.0)	73 (4.8)
Total	1,580 (100)	49 (100)	1,531 (100)

[a]Overall p=0.60

[b]Including eye, ear, sleep, head & neck, neuroendocrine, metabolic, nutritional, neoplasm, device-related complications and genetic/congenital disorders

© 2019 Dayal P et al. *JAMA Network Open.*

eTable 2. Sensitivity Analysis Excluding 39 Patients Who Had Both Telemedicine and In-Person Neurology Appointments

Statistic	Telemedicine (N=339)	In-person (N=3,791)
Hospital encounter rate ratio[a] (95% CI)		
All-cause encounters	0.18 (0.12-0.28)	REF
Neurological encounters	0.22 (0.13-0.37)	REF
Adjusted hospital encounter rate ratio[a,b] (95% CI)		
All-cause encounters	0.42 (0.26-0.68)	REF
Neurological encounters	0.37 (0.21-0.68)	REF

[a]Incident Rate Ratio (IRR) from negative binomial regression with patient's time in the study (years) as an offset

[b]Adjusted for insurance status, median household income, travel time to UCDCH, presence of a complex chronic condition and neurology clinic diagnosis category (similar to Table 4)

© 2019 Dayal P et al. *JAMA Network Open.*

eTable 3. Comparison of Hospital Encounter Frequencies and Rates in Matched Telemedicine and In-Person Patient Cohorts

Statistic	Matched factor			
	Time to UCDCH in minutes[a]		Time to Outpatient Neurology Clinic in minutes[b,c]	
	Telemedicine (N=187)	In-person (N=187)	Telemedicine (N=378)	In-person (N=378)
All-cause encounters, N	52	53	68	369
Neurological encounters, N	34	29	47	211
Total person-years in cohort, mean (SD)	744.3 (31.1)	598.2 (37.7)	1,341.8 (42.2)	1,801.2 (51.4)
Hospital encounter rate ratio (95% CI)				
All-cause encounters	0.59 (0.27-1.29)	REF	0.22 (0.13-0.37)	REF
Neurological encounters	0.70 (0.31-1.55)	REF	0.26 (0.14-0.47)	REF
Adjusted hospital encounter rate ratio[d] (95% CI)				
All-cause encounters	0.58 (0.26-1.30)	REF	0.19 (0.04-0.83)	REF
Neurological encounters	0.79 (0.33-1.92)	REF	0.14 (0.02-0.82)	REF

[a]Mean travel time to UCDCH (minutes): telemedicine cohort=153.4 (SD 46.7), in-person cohort=153.4 (SD 47.2)

[b]Mean travel time to neurology clinic (minutes): telemedicine cohort=20.6 (SD 24.4), in-person cohort=23.5 (SD 23.1)

[c]Inpatient admissions only

[d]Adjusted for insurance status, median household income, travel time to UCDCH (for time to neurology clinic-matched sample only), presence of a complex chronic condition and neurology clinic diagnosis category.

© 2019 Dayal P et al. *JAMA Network Open.*
